# The Big Five Personality Traits and Brain Arousal in the Resting State

**DOI:** 10.3390/brainsci11101272

**Published:** 2021-09-26

**Authors:** Philippe Jawinski, Sebastian Markett, Christian Sander, Jue Huang, Christine Ulke, Ulrich Hegerl, Tilman Hensch

**Affiliations:** 1 Department of Psychology, Humboldt-Universität zu Berlin, 10099 Berlin, Germany; sebastian.markett@hu-berlin.de; 2 LIFE—Leipzig Research Center for Civilization Diseases, University of Leipzig, 04103 Leipzig, Germany; Christian.Sander@medizin.uni-leipzig.de (C.S.); christine.ulke@medizin.uni-leipzig.de (C.U.); Ulrich.Hegerl@kgu.de (U.H.); Tilman.Hensch@medizin.uni-leipzig.de (T.H.); 3 Depression Research Centre, German Depression Foundation, 04109 Leipzig, Germany; 4 Department of Psychiatry and Psychotherapy, University of Leipzig Medical Center, 04103 Leipzig, Germany; Jue.Huang@medizin.uni-leipzig.de; 5 Department of Psychiatry and Psychotherapy, University Hospital Frankfurt, 60323 Frankfurt, Germany; 6 Department of Psychology, IU International University of Applied Science, 99084 Erfurt, Germany

**Keywords:** arousal, Big Five, EEG, resting state, VIGALL, extraversion, neuroticism, impulsivity

## Abstract

Based on Eysenck’s biopsychological trait theory, brain arousal has long been considered to explain individual differences in human personality. Yet, results from empirical studies remained inconclusive. However, most published results have been derived from small samples and, despite inherent limitations, EEG alpha power has usually served as an exclusive indicator for brain arousal. To overcome these problems, we here selected N = 468 individuals of the LIFE-Adult cohort and investigated the associations between the Big Five personality traits and brain arousal by using the validated EEG- and EOG-based analysis tool VIGALL. Our analyses revealed that participants who reported higher levels of extraversion and openness to experience, respectively, exhibited lower levels of brain arousal in the resting state. Bayesian and frequentist analysis results were especially convincing for openness to experience. Among the lower-order personality traits, we obtained the strongest evidence for neuroticism facet ‘impulsivity’ and reduced brain arousal. In line with this, both impulsivity and openness have previously been conceptualized as aspects of extraversion. We regard our findings as well in line with the postulations of Eysenck and consistent with the recently proposed ‘arousal regulation model’. Our results also agree with meta-analytically derived effect sizes in the field of individual differences research, highlighting the need for large (collaborative) studies.

## 1. Introduction

Over the past decades, a substantial body of research has focused on the relationship between individual differences in human personality and the underlying biological mechanisms. Aside from general interest to identify the biological factors that explain the great diversity in human behavior, research in this field has been motivated by theoretical concepts and empirical evidence linking personality traits to mental health outcomes [1,2]. Beyond this, personality traits have been proposed to constitute vulnerability factors for mental diseases, and affective disorders in particular [3,4,5,6,7,8]. On this account, elucidating the biological basis of personality has not only been argued to provide valuable insights into the etiology of psychiatric diseases, but may also have important implications for identifying at-risk individuals, initiating early preventions, and tailoring treatments.

One of the most prominent traits approaches to describe and measure the structure of human personality is the Five-Factor Model (FFM) [9,10]. The FFM is a taxonomy that strives for an economic description of the whole range of individual differences in personality utilizing five overarching factors. These ‘Big Five’ personality traits encompass openness to experience, conscientiousness, extraversion, agreeableness, and neuroticism. With some limitations, the five-factor structure of personality has been shown to generalize across languages and cultures and has been argued to be partly based on innate biological factors [11,12,13]. Evidence from twin studies and genome-wide complex trait analyses suggests that a substantial proportion of the Big Five variance is accounted for by genetics [14,15,16], with molecular genetic studies indicating a highly polygenic architecture [17,18]. However, the biological mechanisms that bridge the effects of genetic variation on human personality remain elusive. To provide an explanatory biological basis of human personality, various neuropsychological trait theories have been postulated, with Eysenck’s Arousal-Activation Theory of Extraversion and Neuroticism having attracted particular attention [19,20].

Eysenck’s Arousal-Activation Theory builds upon the early 1960s’ psychophysiological activation theories, according to which the ascending reticular activation system (ARAS) regulates central nervous system arousal [21,22]. Eysenck distinguishes two components of his conceptual nervous system: the reticulo–cortical brain system (i.e., ARAS) and the reticulo-limbic visceral brain system (VBS) [23]. Excitation of the ARAS by incoming stimuli is referred to as ‘arousal’, whereas the excitation of the VBS by emotional stimuli is referred to as ‘activation’. An increase in activation has arousing effects, while arousal may also occur without activation (i.e., a unidirectional relationship). Eysenck postulated that extraverted individuals possess, on average, relatively low habitual levels of arousal in the resting state, which he traces back to a higher ARAS activation threshold [20]. As a compensatory mechanism, they engage in arousal-enhancing behavior by seeking human interactions as well as novelty, change, and excitement. In comparison, Eysenck describes neurotic individuals as emotionally hypersensitive, which he attributes to a lower activation threshold of the VBS [20]. According to Eysenck, individuals with high levels of neuroticism are more susceptible to stress and show a prolonged autonomic stress response.

The Arousal-Activation Theory has served as a theoretical framework in numerous empirical studies [23,24]. Eysenck himself referred to the alpha range of the human Electroencephalogram (EEG) as the standard measure of arousal [23]. In line with Eysenck’s postulations, a number of studies demonstrated higher resting-state EEG alpha power (indicating lower brain arousal) in extroverted relative to introverted individuals [25,26,27,28,29]. Several other studies failed to provide supportive evidence [30,31,32,33]. In addition, some investigators used EEG beta power as a brain arousal indicator and revealed both supporting and opposing evidence [28,33,34]. In sum, empirical investigations addressing the link between extraversion and brain arousal have provided only inconsistent evidence for Eysenck’s postulations [35]. This inconsistency may be explained by both the insufficient sample size of former studies and the questionable validity of alpha power alone to reflect brain arousal. As we reviewed elsewhere [36], an assessment of arousal by EEG frequencies should consider several aspects, including the high interindividual variability of amplitude and frequency characteristics of resting EEG, the fact that theta and alpha both not only indicate arousal, but also cognitive processes, the effect of opening and closing the eyes, and the temporal dynamics of arousal. The EEG- and EOG-based VIGALL outlined below has the aim to overcome several of these problems of arousal assessment.

A few studies also reported on the relationship between neuroticism and arousal. Based on Eysenck’s postulations, researchers have argued that the habitual level of arousal may tend to be higher in participants scoring high on neuroticism [37]. Consistent with this assumption, investigations in laboratory settings—including resting-state assessments—have previously been shown to elicit an arousal-enhancing ‘first day in lab effect’ [38] similar to the ‘first-night’ effect in sleep medicine [39]. This may especially affect individuals with high levels of neuroticism, who have been proposed to be more vulnerable to stress. Notably, enhanced arousal levels in neurotic individuals would also tie in with the substantial genetic overlap demonstrated between neuroticism and major depression [16,40], with the latter having repeatedly been linked to enhanced and ‘hyperstable’ brain arousal levels in the resting state [41,42,43,44,45,46,47,48]. Despite these converging lines of research, available EEG studies have not yet provided supportive evidence for an association between brain arousal and neuroticism [25,26,29,31]. Again, it should be noted that besides the questionable validity of EEG alpha power as the sole arousal indicator, the vast majority of published results on both neuroticism and extraversion have been derived from small samples with fewer than 100 participants.

In comparison to previous approaches that predominantly used EEG alpha power as an indicator for brain arousal, the above-mentioned studies that demonstrated a link between brain arousal and depression made use of the Vigilance Algorithm Leipzig (VIGALL), an EEG- and EOG-based analysis tool that utilizes low-resolution electromagnetic tomography [49]. Note that the term EEG-vigilance is used for the assessment (operationalization) of brain arousal by EEG. VIGALL is typically applied to fifteen to twenty-minute eyes-closed resting-state recordings and incorporates information on the cortical distribution of the frequency bands alpha, delta, and theta. Beyond this, VIGALL features adaptive procedures that account for the individual differences in alpha peak frequency and EEG total power. Primarily, VIGALL was developed for investigating arousal disturbances in psychiatric samples and to objectively test the assumptions of the ‘arousal regulation model of affective disorders and attention-deficit hyperactivity disorder’ [50]. Similar to Eysenck’s theory, the arousal regulation model postulates that depressive- and manic-like behavior partly reflects an autoregulatory attempt to reduce and enhance habitual high and low arousal levels, respectively. A particular emphasis is put on the regulation of arousal, which is postulated to be unstable in clinical syndromes such as ADHD and mania and is expressed, at the behavioral level, in hyperactivity and sensation seeking (similar to the behavior frequently observed in overtired children). Major depression, in contrast, is postulated to be characterized by enhanced and hyperstable arousal, which is behaviorally expressed in avoidance of external stimulation. By applying VIGALL, a number of empirical studies addressing arousal in depressive, bipolar, and ADHD patients have provided supportive evidence for the assumptions of the arousal regulation model [41,42,43,51,52]. In addition, VIGALL has been validated in an fMRI and PET study [53,54], against evoked potentials and parameters of the autonomous nervous system [55,56,57], against the Multiple Sleep Latency Test [58], and in a large study addressing the agreement with subjective ratings [59]. These previous encouraging results raise the question, whether the application of VIGALL may leverage investigations on the role of arousal in human personality.

Against this background, we here sought to examine the relationship between the Big Five personality traits and brain arousal in the resting state. In comparison to previous EEG studies, we did not assess arousal by alpha power, whose validity has been questioned [36], but by making use of the validated EEG- and EOG-based analysis tool VIGALL. In accordance with prior theoretical and empirical indications, we hypothesized that brain arousal is negatively associated with the personality trait extraversion and positively associated with neuroticism. Notably, each Big Five personality trait has been demonstrated to genetically overlap with psychiatric disorders [16], and each of the respective psychiatric disorders has been proposed to possess arousal-related pathophysiologies [50]. On this account, we here examined the potential associations between brain arousal and each Big Five personality trait. Given the relatively weak effect sizes in personality and individual differences research [60,61], we considered a sample of several hundreds of participants to derive our estimates. In this vein, we sought to contribute empirical evidence to the so-far unresolved issue of whether basic personality dimensions are reflected in habitual levels of arousal.

## 2. Materials and Methods

In the following sections, we report how we determined our sample size, all data exclusions (if any), all manipulations, and all measures in the study [62]. Our statistical analysis scripts have been made publicly available on GitHub (https://github.com/pjawinski/bigv, accessed on 22 September 2021).

### 2.1. Sample

Participants were drawn from the LIFE-Adult study, a population-based cohort study of 10,000 inhabitants of the city of Leipzig, Germany [63]. The scope of LIFE-Adult is to examine prevalences, genetic predispositions, and lifestyle factors of civilization diseases. All participants underwent a comprehensive medical assessment program and completed various psychological surveys. We considered participants with available resting-state EEG and NEO Personality Inventory data (562 participants aged 40–79 years). Of these, we selected participants who reported no current intake of EEG-affecting drugs and had no prior diagnosis of stroke, multiple sclerosis, Parkinson’s disease, epilepsy, skull fracture, cerebral tumor, or meningitis (leaving 533 participants). Based on a structured clinical interview for DSM-IV axis I disorders, we selected participants without a history of psychotic disorders or substance dependence, and who were free of current anxiety and affective disorders (leaving 528 participants). Moreover, EEGs with substantial artifacts (≥15% of all EEG segments) and those showing low-voltage alpha, alpha variant rhythms, or pathological activity were not included. This resulted in N = 468 eligible participants (246 females; mean age: 58.5 years). Participants gave written informed consent and received an expense allowance. All procedures were conducted according to the Declaration of Helsinki and were approved by the Ethics Committee of the University of Leipzig (263-2009-14122009).

### 2.2. Questionnaire

Participants completed the German version of the revised NEO Personality Inventory (NEO-PI-R) [64,65]. The NEO-PI-R is a widely used self-report questionnaire that enables measuring the personality traits neuroticism, extraversion, openness to experience, agreeableness, and conscientiousness. The NEO-PI-R consists of 240 items and ratings are made on a five-point scale ranging from ‘strongly disagree’ to ‘strongly agree’. Item scores are aggregated to the five NEO personality dimensions. The internal consistency (Cronbach’s alpha) of the five overarching factors has been reported to range from 0.87 to 0.92 [64]. Test-retest reliability (1-month interval) has been reported to range from 0.88 to 0.91. Further, the NEO-PI-R allows calculating scores for thirty personality facets, six facets per factor. The internal consistency and test-retest reliability of the facets has been reported to range from 0.53 to 0.85 and 0.48 to 0.90, respectively [64]. NEO personality dimension and facet scores were transformed into sex- and age-normalized T-scores according to the NEO-PI-R manual.

### 2.3. Physiological Data Collection and Processing

Physiological data collection and processing were carried out as previously described [66,67]. EEG assessments were conducted according to a standardized operating procedure. Assessments took place at three-time slots: 8:30 am, 11:00 am, and 1:30 pm. During the twenty-minute resting condition, participants lay on a lounge chair within a light-dimmed sound-attenuated booth. Participants were instructed to close their eyes, relax, and not fight any potential drowsiness. To achieve similar initial levels of arousal activation, all participants completed a brief arithmetic task immediately before the onset of recording. EEGs were derived from 31 electrode positions according to the extended international 10–20 system. Two bipolar electrodes served to record vertical and horizontal eye movements (EOGs). EEGs were recorded against a common average reference with AFz ground. We used a QuickAmp amplifier (Brain Products GmbH, Gilching, Germany) and sampled recordings at 1000 Hz. EEG offline processing was carried out using Brain Vision Analyzer 2.0 (Brain Products GmbH, Gilching, Germany). EEGs were filtered (70 Hz low-pass and 0.5 Hz high-pass with 48 dB/Oct slope, 50 Hz notch) and rectified from eye movement, sweating, cardiac, and muscle artifacts using Independent Component Analysis (ICA). Graph elements (sleep spindles and K-complexes) were manually marked by experienced raters as previously described [59]. Please see the publicly available VIGALL 2.1 manual for further preprocessing details [68].

### 2.4. Assessment of Brain Arousal

The assessment of brain arousal was carried out as described elsewhere [59,66]: EEG-vigilance served as an indicator for brain arousal and was measured using the Brain Vision Analyzer add-on VIGALL 2.1 (https://www.deutsche-depressionshilfe.de/forschungszentrum/aktuellestudien/vigall-vigilance-algorithm-leipzig-2-1, accessed on 22 September 2021) [68]. In brief, based on the cortical distribution and spectral composition of eyes-closed EEG activity, VIGALL assigns one of seven EEG-vigilance stages to each one-second EEG segment. EEG-vigilance stages correspond to active wakefulness (stage 0), relaxed wakefulness (stages A1, A2, A3), drowsiness (stages B1, B2/3), and sleep onset (stage C). Classification criteria put a strong emphasis on the ratio between alpha and the sum of delta and theta band current density measured in different brain regions of interest (occipital, parietal, temporal, and frontal ROIs). For instance, stage A1 is assigned to an EEG segment if (a) no markers indicating sleep onset are present, (b) the alpha band current density in one ROI exceeds the absolute current density threshold for A-stages (threshold set automatically for each individual based on an adaptive procedure), (c) the alpha band current density in the respective ROI exceeds the sum of delta and theta band current density by a factor of two and (d) the occipital alpha-band current density is higher than the parietal, temporal and frontal alpha band current density. Further details on the scoring rules are provided in the VIGALL manual [68] (pp. 28–33). It should be noted that stages A1-3 are characterized by predominant alpha activity, which indicates relatively enhanced brain arousal during eyes-closed resting-state conditions where stages of drowsiness (delta- and theta-activity) and sleep onset (occurrence of K-complexes and sleep spindles) are frequently observed. Therefore, the range of arousal stages implicated in the present study extends traditional approaches where higher EEG alpha power (relaxed wakefulness) has been used as an exclusive indicator for reduced brain arousal. We transformed assigned EEG-vigilance stages into values ranging from 7 (active wakefulness) to 1 (sleep onset) and calculated three outcome variables: mean vigilance, stability score, and slope index. Variable ‘mean vigilance’ provides an estimate for the average level of EEG vigilance during rest. The variables ‘stability score’ and ‘slope index’ particularly focus on the dynamics of EEG-vigilance. Lower scores indicate lower average levels (mean vigilance) and steeper declines (stability score and slope index), respectively, of EEG vigilance. All three outcome variables have been validated, have been found test-retest reliable, and have previously been used as default parameters to summarize complex EEG-vigilance time-courses [38,55,59,69,70].

### 2.5. Statistical Analysis

The internal consistency of the NEO personality dimensions and facets was calculated using SPSS Statistics 27.0 (IBM corp.; Armonk, NY, USA). All frequentist analyses were carried out using Matlab R2020b (The MathWorks Inc., Natick, MA, USA). The nominal level of significance was set at *p* < 0.05 (two-tailed). Further, *p*-values were adjusted by applying the False Discovery Rate (FDR) procedure according to Benjamini and Hochberg [71]. Associations with FDR < 0.05 were regarded as significant after multiple testing corrections. In addition, we sought to derive evidence for the alternate and null hypothesis, respectively, by calculating Bayes factors. Bayes factors reflect the likelihood ratio between the alternate and null hypothesis (BF_10_). Bayesian analyses were conducted with a moderate symmetrical 1/3 beta prior width using package ‘BayesFactor’ [72] in R 4.0.4 [73].

First, we carried out Spearman correlations between the higher-order NEO personality dimensions (sex- and age-normalized T-scores) and the three EEG-vigilance variables (mean vigilance, stability score, and slope index). Next, we generated a permutation-based quantile-quantile plot (qq-plot) to examine whether the distribution of observed *p*-values differs from a random *p*-value distribution under the null hypothesis. On this account, for the set of 15 observed *p*-values (5 NEO personality dimensions ×3 EEG-vigilance variables), one million sets of 15 expected *p*-values were derived from correlations after data permutation. Original correlations within the domain of personality traits and the domain of EEG-vigilance variables were preserved, whereas original correlations between these domains were removed through random shuffling. Subsequently, in order to identify facets that particularly contribute to the observed associations, we conducted exploratory Spearman correlations between the thirty NEO personality facets (sex- and age-normalized T-scores) and the three EEG-vigilance variables. By analogy to the higher-order ‘Big Five’ analyses, we also generated a permutation-based qq-plot for the NEO personality facets. Analyses were repeated with sex, age, and daytime of EEG-assessment serving as covariates.

### 2.6. Statistical Power

Power analyses were conducted using R package pwr (version 1.3-0) [74], with effect sizes quantified as Spearman’s rho (r_S_). Given N = 468 and α = 0.05, power calculations revealed that associations with true effect sizes of r_S_ = 0.052, r_S_ = 0.091, and r_S_ = 0.129 were identified with a chance of 20%, 50% and 80% (1-β), respectively. After Bonferroni-correction (α = 0.0033; resembling the most conservative case where the FDR procedure ends at the smallest observed *p*-value), power calculations revealed that associations with true effect sizes of r_S_ = 0.097, r_S_ = 0.135, and r_S_ = 0.173 were identified with a chance of 20%, 50% and 80% (1-β), respectively. Supplementary Figure S1 shows the probabilities of associations to reach the threshold of significance, given true effect sizes of up to r_S_ = 0.4.

## 3. Results

The descriptive statistics for the five higher-order NEO personality traits and the three EEG-vigilance variables are shown in Table 1. The internal consistency (Cronbach’s alpha) of the five NEO personality dimensions ranged between 0.84 and 0.91 and was thus comparable to previous reports [64]. The NEO personality dimensions were significantly intercorrelated (Supplementary Table S1), with the strongest correlation observed between extraversion and openness to experience (r_S_ = 0.477, *p* = 5 × 10^−28^).

The internal consistency of the NEO personality facets ranged between 0.46 and 0.83 (Cronbach’s alpha and intercorrelations shown in Supplementary Figure S2). Intercorrelations between EEG-vigilance variables reached r_S_ ≥ 0.82 (Supplementary Table S2). Regarding covariates, we observed that younger participants and those who underwent the EEG assessment at later daytime exhibited a lower EEG-vigilance (e.g., mean vigilance; age: r_S_ = 0.168, *p* = 3 × 10^−4^; daytime: r_S_ = −0.155, *p* = 8 × 10^−4^). Although we used to sex- and age-normalized T-scores according to the NEO-PI-R manual, we observed some remaining associations between the NEO personality traits and both sex and age. Detailed association results between covariates and variables of interest are shown in Supplementary Table S3.

### 3.1. Big Five Personality Traits and Brain Arousal

Spearman correlations between the five NEO personality dimensions (sex and age-normalized T-scores) and the three EEG-vigilance variables are shown in Table 2.

**Figure 1:**
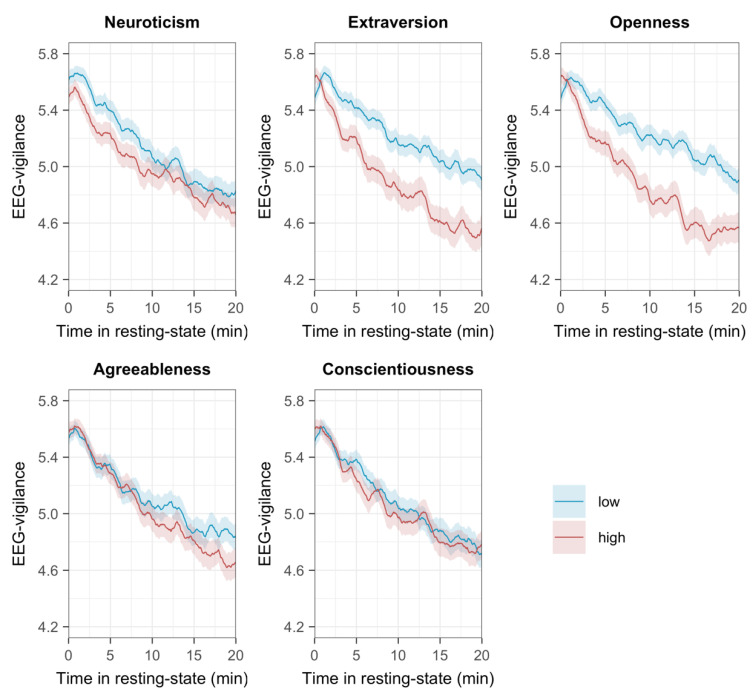
Time-courses of EEG-vigilance during the 20-min eyes-closed resting-state condition stratified by groups scoring low vs. high on the respective Big Five scale (median-split, i.e., participants with scores in the lower vs. upper half of the ascending distribution are compared). Time-courses reflect simple moving averages (± standard errors), i.e., every data point represents an averaged 61-s interval of EEG-vigilance (data point in time ± 30 s). Statistical analyses revealed significant associations between EEG-vigilance and both extraversion and openness to experience.

**Figure 2:**
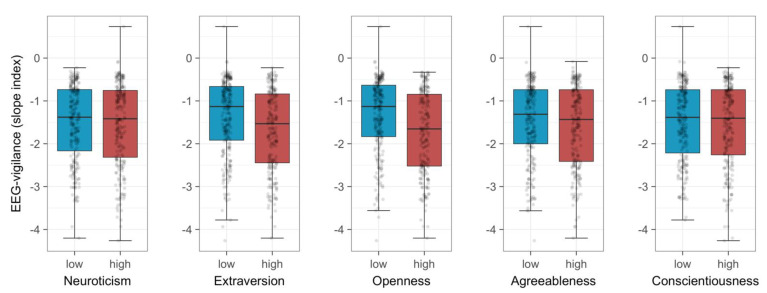
Boxplots showing the differences in EEG-vigilance (as indicated by variable ‘slope index’) as a function of Big Five personality trait. Boxplots are stratified by groups scoring low vs. high on the respective Big Five scale (i.e., participants with scores in the lower vs. upper half of the ascending distribution are compared). Boxes represent the interquartile range (data between the lower and upper quartile), with the horizontal line corresponding to the median. Whiskers extend to the furthest observation within 1.5 times the interquartile range from the lower and upper quartile. Dots represent single observations, jittered horizontally to avoid overplotting. Statistical analyses revealed significant associations between EEG-vigilance variable ‘slope index’ and both extraversion and openness to experience.

Analyses revealed six associations reaching nominal significance (*p* < 0.05). Of these, three remained significant after multiple testing correction (FDR < 0.05). We observed EEG-vigilance to be inversely associated with the degree of extraversion (slope index: r_S_ = −0.137, *p* = 0.003, FDR = 0.023, BF_10_ = 8.35) and openness to experience (stability score: r_S_ = −0.121, *p* = 0.009, FDR = 0.044, BF_10_ = 3.23; slope index: r_S_ = −0.173, *p* = 2 × 10^−4^, FDR = 0.002, BF_10_ = 121.33). Participants who reported higher levels of extraversion and openness to experience, respectively, exhibited lower EEG-vigilance. For illustrative purposes, the time-courses of EEG-vigilance stratified by groups scoring low vs. high on the respective Big Five dimension (median-split) are shown in Figure 1. Boxplots showing the differences in EEG-vigilance (as indicated by variable ‘slope index’) among participants scoring low vs. high on the respective Big Five scale are provided in Figure 2.

**Figure 3:**
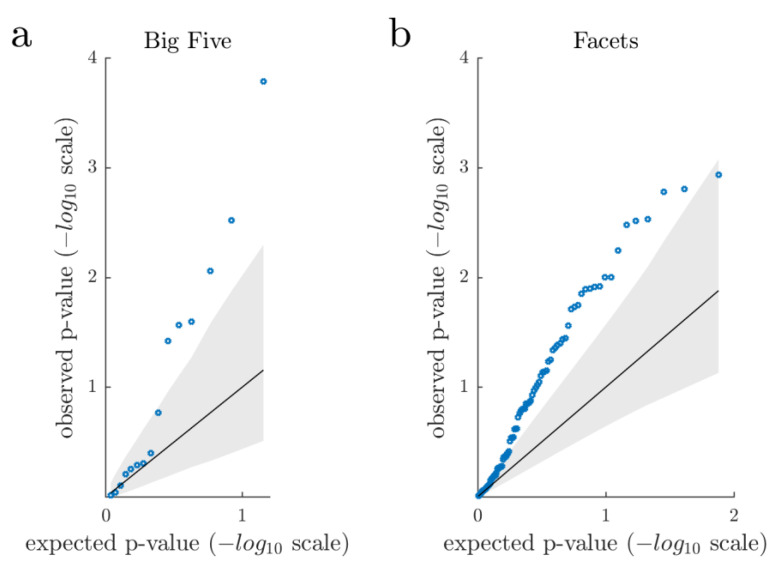
Permutation-based qq-plot showing the observed *p*-values from the association analyses (blue circles) sorted from largest to smallest and plotted against the expected *p*-values under the null hypothesis. The solid diagonal line represents the mean expected *p*-values. The lower and upper bound of the grey area represents the 5th and 95th percentile (−log10 scale) of the expected *p*-values. Quantile-quantile plots show an excess of low *p*-values, suggesting that association analyses revealed overall stronger evidence than expected under the null hypothesis of no effect. (**a**) NEO personality traits (sex and age-normalized T-scores) (**b**) NEO personality facets (sex and age-normalized T-scores).

We repeated our analysis by additionally adjusting sex- and age-normalized T-score correlations by sex, age, and daytime of EEG assessment (Supplementary Table S4). This resulted in three associations reaching nominal significance. Of these, one remained significant after multiple testing correction (in this case the FDR corrected *p*-value is equivalent to the Bonferroni-corrected *p*-value): Participants who reported higher levels of openness to experience exhibited lower EEG-vigilance (slope index: r_S_ = −0.152, *p* = 0.001, FDR = 0.015, BF_10_ = 23.40). To examine whether the distribution of observed *p*-values differs from a random *p*-value distribution, we generated a permutation-based qq-plot that takes into account the dependencies between association tests (Figure 3a).

Figure 3a shows an excess of low *p*-values for the set of tested associations, with the six strongest observed *p*-values exceeding the 95th percentile (−log10 scale) of the computed expected *p*-value distribution. These six observed *p*-values also indicated nominal significance (*p* < 0.05). By comparison, only 1.1% of the one million sets of expected *p*-values contained at least six *p*-values below 0.05 (nominal significance), and 0.2% of the one million sets of expected *p*-values contained at least one *p*-value below 2 × 10^−4^ (that is the lowest observed *p*-value). Overall, the plot indicates that the distribution of observed *p*-values differs from a random *p*-value distribution under the null hypothesis. When additionally adjusting sex- and age-normalized T-score associations by sex, age, and daytime of EEG assessment, only the strongest observed association exceeded the 95th percentile of the expected *p*-value distribution (Supplementary Figure S3a). In this regard, only 1.1% of the one million sets of expected *p*-values contained one *p*-value lower than 0.001 (that is the lowest observed *p*-value).

### 3.2. NEO Personality Facets and Brain Arousal

To further elaborate the nature of the underlying associations, we carried out exploratory Spearman correlations between the 30 NEO facets (sex and age-normalized T-scores) and each of the 3 EEG-vigilance variables. Detailed association results are shown in Supplementary Table S5. In total, 24 out of 90 correlations reached the level of nominal significance. The strongest association was observed for neuroticism facet ‘impulsiveness’ (mean vigilance: r_S_ = −0.150, *p* = 0.001, BF_10_ = 19.88). No other neuroticism facet reached nominal significance. Regarding extraversion, we found nominally significant results for the facets ‘warmth’ (slope index: r_S_ = −0.119, *p* = 0.010, BF_10_ = 2.90), ‘assertiveness’ (slope index: r_S_ = −0.109, *p* = 0.018, BF_10_ = 1.73), ‘activity’ (slope index: r_S_ = −0.114, *p* = 0.014, BF_10_ = 2.14), and ‘positive emotions’ (slope index: r_S_ = −0.116, *p* = 0.012, BF_10_ = 2.43). Regarding openness to experience, we found significant associations for facets ‘fantasy’ (slope index: r_S_ = −0.094, *p* = 0.041, BF_10_ = 0.85), ‘aesthetics’ (slope index: r_S_ = −0.137, *p* = 0.003, BF_10_ = 8.60), ‘feelings’ (slope index: r_S_ = −0.137, *p* = 0.003, BF_10_ = 8.27), ‘actions’ (slope index: r_S_ = −0.109, *p* = 0.018, BF_10_ = 1.68), and ‘ideas’ (slope index: r_S_ = −0.128, *p* = 0.006, BF_10_ = 4.76). We also observed nominally significant results for agreeableness facet ‘tender-mindedness’ (slope index: r_S_ = −0.145, *p* = 0.002, BF_10_ = 14.40) and conscientiousness facet ‘achievement striving’ (slope index: r_S_ = −0.135, *p* = 0.003, BF_10_ = 7.65).

By analogy to the NEO personality dimension analyses, we generated a permutation-based qq-plot to examine whether the distribution of observed *p*-values of the NEO personality facets differs from a random *p*-value distribution (Figure 3b). Again, the qq-plot indicates that association analyses revealed stronger evidence than expected under the null hypothesis of no effect. It should be noted that, consistent with the Big Five results, we observed an attenuation of effect sizes when additionally adjusting sex- and age-normalized T-Score correlations by sex, age, and daytime of EEG-assessment (Supplementary Table S6). The distribution of observed vs. expected *p*-values after adjusting T-Scores is shown in Supplementary Figure S3b, with a large proportion still exceeding the 95th percentile of expected *p*-values.

## 4. Discussion

In this study, we investigated the association between the Big Five personality traits and brain arousal in the resting state. To overcome the limitations of prior studies, we enrolled a large sample and applied an advanced EEG- and EOG-based arousal assessment tool (VIGALL). Our primary analysis suggests that, after multiple testing corrections, brain arousal is negatively associated with the degree of extraversion and openness to experience: Participants who reported higher levels of extraversion and openness to experience, respectively, showed steeper declines of arousal. In addition, when considering all tested associations between the Big Five personality traits and brain arousal, we observed overall stronger effects than expected under the null hypothesis of no effect. This finding was supported by association results of the thirty NEO personality facets. Facet analyses also revealed that the observed associations of the higher-order Big Five traits and brain arousal were not driven by a single facet with a distinct, strong effect but rather appeared to arise from a distributed pattern of associations across several facets. Notably, for the majority of nominally significant associations (*p* < 0.05), Bayesian analysis revealed only anecdotal evidence for the alternate hypothesis (BF_10_ ranging between 1 and 3). In addition, when taking into account potential confounders, we observed a general attenuation of effect sizes, with several associations dropping below the nominal and FDR-corrected level of significance. Further, we did not obtain evidence for an association between brain arousal and neuroticism. Overall, across frequentist and Bayesian analyses and irrespective of accounting for potential confounders or not, we obtained the strongest and most compelling evidence for a link between openness to experience and brain arousal.

To our knowledge, this investigation is the largest EEG study so far addressing the link between brain arousal and the Big Five personality traits. In keeping with this, the statistical power to detect associations in this study was substantially higher when compared to the vast majority of previous investigations, which usually featured a sample size of fewer than 100 participants. Given the present study design and analysis procedure, the achieved statistical power enables us to conclude that neuroticism is unlikely to account for more than 4% (r_S_ ≥ 0.2) of the variance in brain arousal since the probability (1-β) to identify such an effect at *p* < 0.05 exceeded 99.98% (see Supplementary Figure S1 for a power plot). Similarly, extraversion surpassed the FDR corrected but not the Bonferroni-adjusted level of significance, with the latter being reached with a probability above 92% given a true effect size of r ≥ 0.20. Thus, if extraversion and neuroticism are truly associated with brain arousal, correlations are certainly below r = 0.20. This is well in agreement with a study of 708 meta-analytically derived correlations in the field of personality and individual differences research, suggesting a median reported effect size of r = 0.19 [60]. Notably, when considering preregistered studies only, the median effect size has been reported to be even lower (r = 0.12; Schäfer & Schwarz, 2019). Accordingly, to elucidate the biological basis of individual differences in human personality, we believe that there is an urgent need for large (collaborative) studies such as the Genetics of Personality Consortium and ENIGMA-EEG [17,75].

The present study adds empirical results to the ongoing debate of whether extraverted individuals exhibit lower habitual levels of brain arousal. Consistent with the theoretical assumptions, our primary analyses provided supportive evidence for a negative correlation between extraversion and arousal. Nevertheless, there remain some reservations that we would like to outline. First, although we used to sex- and age-normalized T-scores, we still observed associations between the NEO personality scores and both sex and age (Supplementary Table S3). After considering sex and age as additional covariates, the observed associations between extraversion and brain arousal did not remain significant after multiple testing corrections. Hence, the present results do not provide stringent support for extraversion to share unique variance with brain arousal beyond the effects of sex and age. Second, Bayesian analyses provided only anecdotal to moderate evidence for the proposed link. This may partly be explained by the selected priors. Here, we used a symmetrically scaled 1/3 beta prior, which is the default setting of the R package ‘BayesFactor’ [72]. This prior corresponds to the expectation that with an 80% probability the true effect falls in between r = −0.5 and r = 0.5. However, in light of the reported effect sizes in the field of individual differences research [60,61], this prior width might still be considered too wide, and resulting Bayes factors may thus show some bias towards favoring the null hypothesis. Notably, the software package JASP, which is widely used in the social and behavioral sciences, implicates an even more naive uniform before the default setting [76]. Hence, given the rising popularity of Bayesian analyses in the life sciences, we feel that the selection of adequate prior widths may be one crucial topic of future scientific debate. Taken together, we here find some evidence supporting Eysenck’s postulations concerning the link between extraversion and brain arousal, but the observed effect strongly suggests that even larger sample sizes are required to establish reliable associations that withstand a rigorous control for potential confounders. An elaborated a priori knowledge of the expected effect sizes may further increase the study power.

Although we did not obtain evidence for a link between brain arousal and neuroticism, exploratory analyses revealed indications for a negative association with neuroticism facet ‘impulsiveness’. This result was the most compelling among all facet associations and remained significant after multiple-testing correction. Interestingly, impulsiveness showed relatively low correlations with the other neuroticism facets and, in contrast to them, correlated positively with facets of extraversion and openness to experience (Supplementary Figure S2). The observation of low habitual arousal levels in individuals exhibiting impulsive behavior is well in line with previously proposed concepts [19,50,77]. It has been argued that the impulsivity facet, which was only inherent in Eysenck’s earlier extraversion scales, was most important for the association with reduced arousal [78,79,80]. Whereas the first extraversion scales have been made up by items on impulsivity and sociability, impulsiveness items have largely been removed later on. This was suggested to explain inconsistent findings across different studies [78,79,80]. In the same vein, other studies showed that preference for stimulation and cognitive performance, both considered as arousal correlates, were more related to extraversion’s impulsivity facet than to its sociability facet [79].

In comparison to neuroticism facet ‘impulsiveness’, our analyses did not reveal indications for a link between neuroticism facet ‘depression’ and brain arousal. By applying the Vigilance Algorithm Leipzig, several previous studies provided supportive evidence for an association between clinical depression and enhanced brain arousal in the resting state [41,42,43,44,45,46,47,48,51]. Although the present study included participants without a current depression diagnosis, it can be assumed that alterations in brain arousal occur in both the normal and pathological range of human behavior. This is consistent with the view that personality traits and psychopathology are no distinct entities, but may rather manifest along a common spectrum of functioning [81]. This argumentation also ties in with the postulations of the Research Domain Criteria Project (RDoC), according to which mental diseases can be considered to fall along multiple continuous trait dimensions, with traits ranging from normal to the extreme [82]. Intriguingly, the RDoC project considers ‘arousal and regulatory systems’ as one out of five fundamental domains to describe and classify psychiatric disorders. In the present study, the lack of evidence for an association between brain arousal and ‘depression’ may be explained by lower effect sizes among healthy participants relative to the study of healthy control vs. in-patient samples.

Across all analyses, we obtained the strongest evidence for an association between the Big Five personality trait openness to experience and brain arousal. Bayes factors indicated ‘extreme evidence’ (BF_10_ > 100) and ‘strong evidence’ (BF_10_ ranging from 10 to 30) for this link, respectively, depending on whether we considered zero-order correlations or whether sex, age, and daytime of EEG assessment served as covariates. Previous investigations have shown that openness to experience positively correlates with sensation seeking [83]. Further, openness to experience has been reported to positively correlate with extraversion [64], which is also shown in the present dataset (Supplementary Table S1). Due to their strong correlation, extraversion and openness to experience constitute one common factor in the Big Four Modell by Becker [84]. On this account, we regard the present findings of lower arousal levels in participants scoring high on openness to experience as consistent with the concepts of Eysenck [19] and Zuckerman [77].

Interestingly, the two nominal significant associations of arousal with facets beyond the extraversion/openness factor and impulsivity, are also quite plausible. First, the agreeableness facet ‘tender-mindedness’ correlated negatively with arousal. Tender-mindedness shows substantial association with extraversion facet ‘warmth’ and also with openness facets [64]. Secondly, the conscientiousness facet ‘achievement striving’ is associated with the extraversion facets ‘gregariousness’ and ‘assertiveness’ [64] and is indeed a common part of extraversion concepts in biological personality theories [85]. Thus, all facets associated with arousal comprise aspects of the extraversion construct.

Our study poses some limitations that need to be addressed. First, our participants were, on average, 58 years old, and reported association strengths may not generalize across other age groups. In particular, we previously observed a general tendency towards stronger effect sizes for arousal associations among the younger age groups [59]. This might be explained by the higher EEG total power in younger adults and a possibly related higher accuracy of EEG-vigilance classifications. In addition, our participants demonstrated lower T-values in neuroticism and openness, higher T-values in conscientiousness and agreeableness, as well as lower variances across the five personality traits when compared to German normative data (see Table 1 in Section 3). Potentially stronger effect sizes might be found in more heterogeneous samples with greater variability in personality traits. Further, we here addressed the relationship between individual differences in personality and habitual levels of brain arousal utilizing an EEG resting-state paradigm. However, one major emphasis of Eysenck’s theory is put on the differential performance of extroverts and introverts as a function of arousal-enhancing situational factors [20]. Thus, an interesting future direction might be the use of VIGALL in experimental studies with behavioral performance outcomes (e.g., as done previously by Huang et al. [55]) while taking into account the ‘hedonic tone of an individual’, i.e., the preferred level of excitation [25]. Moreover, it should be noted that we made use of a questionnaire (NEO-PI-R) that is based on the Big Five personality model, while most previous EEG studies in this field used the Eysenck personality inventories. Although method heterogeneity generally hampers direct comparisons, previous studies have reported that both types of instruments, i.e., the Big Five and Eysenck Personality inventories, strongly overlap in item content and show high correlations between sum scores of extraversion and neuroticism [86,87,88,89]. Furthermore, we would like to emphasize that our study design involved an eyes-closed EEG assessment, while previous studies employed eyes-opened [26], eyes-closed [27,29], or both eyes-closed and eyes-opened conditions [28,31,32,33,34]. Lastly, it should be noted that the present study sought to answer the question of whether brain arousal but not the EEG, in general, is predictive for basic personality traits. Stronger associations may be derived by the application of machine learning models trained on the EEG to directly predict human personality (for example, see Li et al. [90]).

## 5. Conclusions

To the best of our knowledge, this study is the largest EEG study so far addressing the relationship between personality traits and habitual levels of brain arousal. Concerning Eysenck’s Arousal Activation Theory, our results provide some support for extraversion and no support for neuroticism to be linked to brain arousal. Intriguingly, Bayesian and frequentist analyses revealed convincing evidence for a link between openness to experience and lower levels of brain arousal. In addition, among the lower-order personality traits, we obtained evidence for neuroticism facet ‘impulsivity’ and reduced brain arousal. We regard these findings as well in line with the postulations of Eysenck and consistent with the assumptions of the arousal regulation model. In total, the present study results agree with meta-analytically derived effect sizes in the field of personality and individual differences research, highlighting the need for large (collaborative) studies.

## Figures and Tables

**Table 1:** Descriptive statistics of NEO-PI-R scores and VIGALL 2.1 variables of EEG-vigilance.

N = 468	Cronbach’s α	Mean	SD	Q1	Q2	Q3	Min	Max	Skew	Kurt
NEO-PI-R										
Neuroticism	0.906	44.95	8.77	39.00	45.00	52.00	20.00	71.00	−0.23	−0.29
Extraversion	0.899	51.69	9.83	45.00	52.00	58.00	21.00	79.00	−0.02	0.18
Openness	0.868	46.85	8.45	41.00	46.00	51.00	20.00	72.00	0.32	0.32
Conscientiousness	0.836	53.02	8.87	47.00	52.00	59.00	26.00	80.00	0.10	0.09
Agreeableness	0.881	54.73	9.04	48.00	54.00	61.00	30.00	80.00	0.30	0.03
EEG-vigilance										
Mean vigilance	-	5.09	1.06	4.43	5.40	5.89	1.93	6.76	−0.90	−0.03
Stability score	-	9.20	4.19	6.00	9.00	13.00	1.00	14.00	−0.58	−0.94
Slope index	-	−1.52	0.92	−2.26	−1.38	−0.74	−4.26	0.73	−0.57	−0.58

SD: standard deviation, Q1: quartile 1, Q2: quartile 2 (median), Q3: quartile 3, Min: minimum observed value; Max: maximum observed value; Skew: skewness, Kurt: excess kurtosis.

**Table 2:** Spearman correlations between NEO personality dimensions (T-Scores) and EEG-vigilance variables.

*N* = 468	Mean Vigilance					Stability Score						Slope Index					
	rho	*p*		FDR	BF_10_	rho	*p*		FDR		BF_10_	rho	*p*		FDR		BF_10_
Neuroticism	−0.063	0.170		0.365	0.27	−0.030	0.515		0.766		0.13	−0.002	0.972		0.972		0.11
Extraversion	−0.104	0.025	*	0.082	1.29	−0.096	0.038	*	0.095		0.91	−0.137	0.003	*	0.023	**	8.35
Openness To Experience	−0.102	0.027	*	0.082	1.20	−0.121	0.009	*	0.044	**	3.23	−0.173	2 × 10^−4^	*	0.002	**	121.33
Agreeableness	−0.027	0.561		0.766	0.13	−0.012	0.791		0.913		0.11	−0.032	0.496		0.766		0.14
Conscientiousness	−0.023	0.624		0.780	0.12	−0.005	0.913		0.972		0.11	−0.039	0.399		0.748		0.15

FDR: False Discovery Rate according to Benjamini and Hochberg; BF_10_ Bayes factor showing the likelihood ratio between the alternate and null hypothesis (1/3 beta prior width). * *p* < 0.05 (two-tailed nominal significance). ** FDR < 0.05 (*p*-value corrected for all tested associations using FDR method).

## Data Availability

Restrictions apply to the availability of these data. Data were obtained from the Leipzig Research Center for Civilisation Diseases. All data and samples of LIFE are the property of the University of Leipzig and are subject to the Law for the Protection of Informal Self-Determination in the Free State of Saxony (Saxon Data Protection Act). The use of data can be requested through the LIFE office (https://life.uni-leipzig.de/, accessed on 22 September 2021). We made our statistical analysis code available on GitHub at https://github.com/pjawinski/bigv (accessed on 22 September 2021).

## Supplementary Materials

The following are available online at https://www.mdpi.com/article/10.3390/brainsci11101272/s1, Figure S1: Power analysis results, Figure S2: NEO personality facets (T-scores)—Cronbach’s Alpha and intercorrelations, Figure S3: Permutation-based qq-plot of observed vs. expected p-values for NEO personality traits and facets (T-Scores) after adjusting for covariates, Table S1: NEO personality dimensions (T-scores)—Cronbach’s Alpha, Table S2: Intercorrelations between EEG-vigilance variables, Table S3: Correlations of the covariates sex, age, and daytime of EEG assessment with NEO personality traits (T-scores) and EEG-vigilance and intercorrelations, Table S4: Partial Spearman correlations between NEO personality dimensions (T-Scores) and EEG-vigilance variables, Table S5: Spearman correlations between NEO personality facets (T-Scores) and EEG-vigilance variables, Table S6: Partial Spearman correlations between NEO personality facets (T-Scores) and EEG-vigilance variables.
