# Supplementary material for: The Big Five Personality Traits and Brain Arousal in the Resting State
Source: Brain Sci. 2021 Sep 26;11(10):1272. doi: 10.3390/brainsci11101272 (PMC8533901; doi:10.3390/brainsci11101272)
Supplement: Supplementary file 1 [file brainsci-11-01272-s001.zip › brainsci-1385492-supplementary.pdf]

Supplementary material to: The Big Five personality traits and brain arousal in the resting state

Power analysis and intercorrelations

Supplementary Fig. S1	Power analysis results
Supplementary Table S1	NEO personality dimensions (T-scores) - Cronbach's Alpha and intercorrelations
Supplementary Fig. S2	NEO personality facets (T-scores) - Cronbach's Alpha and intercorrelations
Supplementary Table S2	Intercorrelations between EEG-vigilance variables
Supplementary Table S3	Correlations of the covariates sex, age, and daytime of EEG assessment with NEO personality traits (T-scores) and EEG-vigilance

Associations between NEO personality traits and EEG-vigilance

Supplementary Fig. S3	Permutation-based qq-plot of observed vs. expected p-values for NEO personality traits and facets (T-Scores) after adjusting for covariates
Supplementary Table S4	Partial Spearman correlations between NEO personality dimensions (T-Scores) and EEG-vigilance variables
Supplementary Table S5	Spearman correlations between NEO personality facets (T-Scores) and EEG-vigilance variables
Supplementary Table S6	Partial Spearman correlations between NEO personality facets (T-Scores) and EEG-vigilance variables

Power analysis and psychometric properties

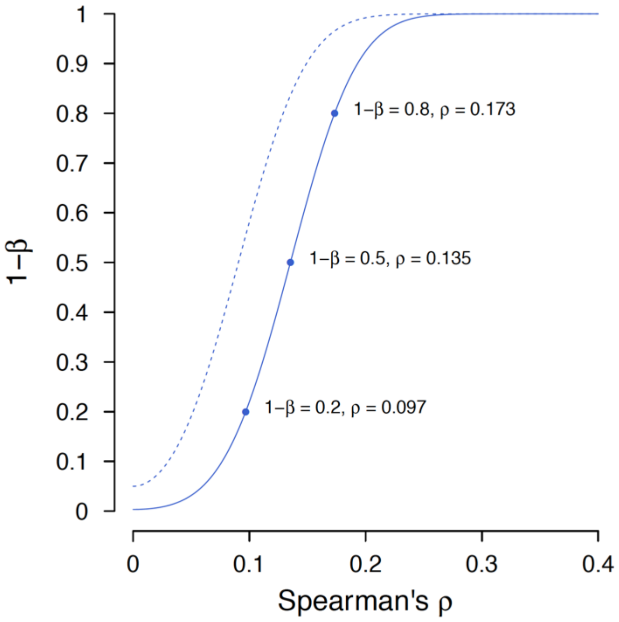

Fig. S1 Power analysis results showing the probability (1-β) of associations to surpass the threshold of significance given true effect sizes ranging between ρ = 0.0 and ρ = 0.4 (with N = 468). The dotted curve shows the probability to reach nominal significance (α = 0.05, two-tailed). The solid curve shows the probability to reach the Bonferroni-corrected level of significance (α = 0.05/15, two-tailed). Power analysis was conducted using R package pwr v1.3-0 (Champely, 2020).

Table S1 NEO personality traits (T-Scores) – Cronbach's α and intercorrelations

N = 468	N		E		O		A		C	
	r_S	p	r_S	p	r_S	p	r_S	p	r_S	p
N Neuroticism	.906	-								
E Extraversion	-.337	7E-14*	.899	-						
O Openness	-.249	5E-8*	.477	5E-28*	.868	-				
A Agreeableness	-.190	4E-5*	-.069	.136	.071	.127	.836	-		
C Conscientiousness	-.440	1E-23*	.333	1E-13*	.121	.009*	.200	1E-5*	.881	-

Results show Spearman correlations. Values of the main diagonal reflect the internal consistency (Cronbach's α).

* $p < .05$ (two-tailed nominal significance)

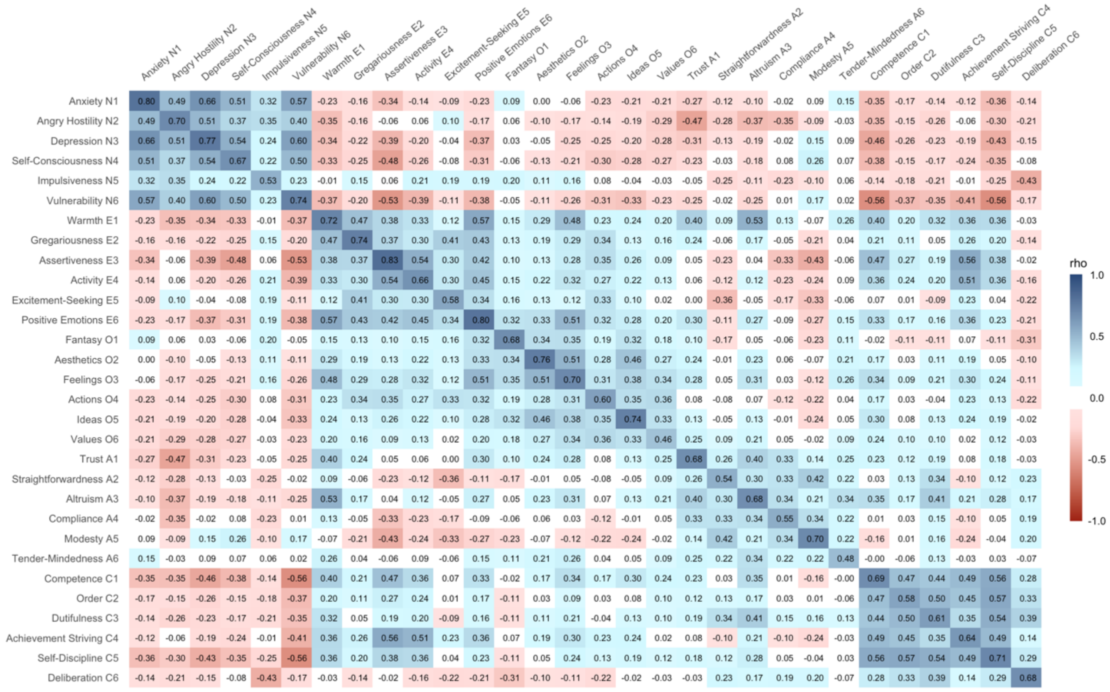

Fig. S2 NEO personality facets - Cronbach's α and intercorrelations. Only cells containing correlations with nominal significance ($p < 0.05$) have been assigned with colors of the blue and red color palette. Values of the main diagonal reflect the internal consistency as estimated using Cronbach's α. We provide an interactive version of this plot with further details in our GitHub repository (https://github.com/pjawinski/bigv).

Table S2 Intercorrelations between EEG-vigilance correlations

$N = 468$	Mean vigilance		Stability score		Slope index	
	r_S	p	r_S	p	r_S	p
Mean vigilance	1.000	-				
Stability score	.821	1E-115*	1.000	-		
Slope index	.820	5E-115*	.881	9E-154*	1.000	-

Results show Spearman correlations.

* $p < .05$ (two-tailed nominal significance)

Table S3 Correlations of sex, age, and daytime of EEG assessment with NEO personality traits (T-scores) and EEG-vigilance variables

$N = 468$	Sex		Age		Daytime	
	r_S	p	r_S	p	r_S	p
**EEG-vigilance**						
Mean vigilance	.021	.648	.168	3E-4*	-.155	8E-4*
Stability score	.030	.523	.178	1E-4*	-.197	2E-5*
Slope index	.084	.069	.191	3E-5*	-.167	3E-4*
**NEO personality dimensions**						
Neuroticism	.064	.164	-.028	.539	.017	.722
Extraversion	-.161	5E-4*	-.074	.111	.068	.143
Openness	-.076	.102	-.084	.070	.012	.792
Agreeableness	.009	.840	-.074	.109	-.070	.131
Conscientiousness	-.164	4E-4*	.080	.082	-.020	.662
**NEO personality facets**						
Neuroticism						
N1 Anxiety	.028	.539	-.024	.606	.010	.825
N2 Angry Hostility	.076	.099	.012	.803	.040	.383
N3 Depression	.047	.314	.025	.597	.018	.692
N4 Self-Consciousness	.085	.067	-.060	.199	-.048	.298
N5 Impulsiveness	-.050	.279	.012	.801	.048	.296
N6 Vulnerability	.114	.013*	-.066	.154	-.039	.400
Extraversion						
E1 Warmth	-.115	.012*	-.093	.044*	.087	.059
E2 Gregariousness	-.032	.484	-.140	.002*	.009	.842
E3 Assertiveness	-.078	.092	.024	.598	.087	.060
E4 Activity	-.142	.002*	-.014	.765	.067	.148
E5 Excitement-Seeking	-.095	.040*	-.050	.279	.020	.659
E6 Positive Emotions	-.233	4E-7*	-.061	.190	.042	.366
Openness						
O1 Fantasy	-.060	.192	-.065	.159	.005	.908
O2 Aesthetics	-.049	.289	-.055	.237	.038	.411
O3 Feelings	-.108	.020*	-.101	.029*	.041	.375
O4 Actions	-.039	.398	-.049	.288	.045	.333
O5 Ideas	-.018	.702	-.010	.833	-.030	.521
O6 Values	-.035	.452	.000	.998	-.069	.137
Agreeableness						
A1 Trust	-.035	.452	-.074	.112	-.008	.861
A2 Straightforwardness	.038	.413	-.015	.754	-.036	.431
A3 Altruism	-.103	.026*	-.069	.137	-.037	.423
A4 Compliance	.123	.008*	-.024	.604	-.055	.239
A5 Modesty	.006	.897	.031	.505	-.073	.115
A6 Tender-Mindedness	-.002	.969	-.165	4E-4*	-.042	.359
Conscientiousness						
C1 Competence	-.129	.005*	.040	.384	.026	.581
C2 Order	-.119	.010*	.087	.061	-.048	.299
C3 Dutifulness	-.184	6E-5*	-.013	.775	-.021	.653
C4 Achievement Striving	-.129	.005*	.062	.180	.052	.264
C5 Self-Discipline	-.093	.045*	.039	.400	-.050	.285
C6 Deliberation	-.050	.278	.103	.026*	-.052	.261

Results show Spearman correlations. Sex was coded as male = 1 and female = 2.

* $p < .05$ (two-tailed nominal significance)

Associations between NEO personality traits and EEG-vigilance

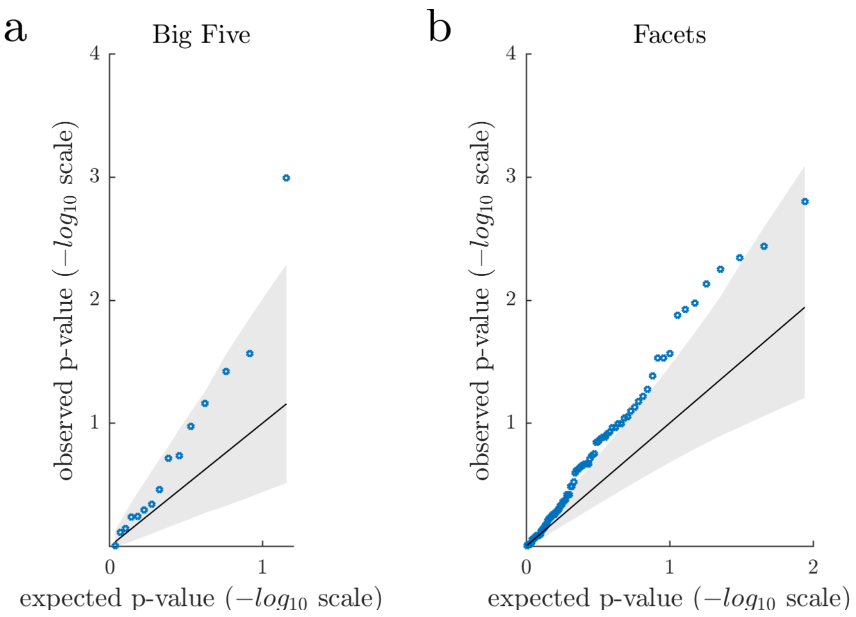

Fig. S3 Permutation-based qq-plot showing the observed p-values from the association analyses (blue circles) sorted from largest to smallest and plotted against the expected p-values under the null hypothesis. The solid diagonal line represents the mean expected p-values. The lower and upper bound of the grey area represent the 5th and 95th percentile (-log10 scale) of the expected p-values. **(a)** NEO personality traits (sex and age-normalized T-scores) adjusted by sex, age, and time of EEG assessment. **(b)** NEO personality facets (sex and age-normalized T-scores) adjusted by sex, age, and time of EEG assessment.

Table S4 Partial Spearman correlations between NEO personality dimensions (T-Scores) and EEG-vigilance variables

$N = 468$	Mean vigilance				Stability score				Slope index			
	r_S	p	FDR	BF_{10}	r_S	p	FDR	BF_{10}	r_S	p	FDR	BF_{10}
Neuroticism	-.060	.193	.414	0.25	-.026	.577	.721	0.13	.000	.997	.997	0.11
Extraversion	-.075	.106	.318	0.40	-.062	.184	.414	0.26	-.096	.038*	.189	0.92
Openness	-.084	.069	.260	0.56	-.103	.027*	.189	1.23	-.152	.001*	.015**	23.40
Agreeableness	-.026	.572	.721	0.13	-.013	.773	.828	0.11	-.031	.508	.721	0.13
Conscientiousness	-.035	.456	.721	0.14	-.017	.721	.828	0.12	-.044	.345	.647	0.17

Effects of sex, age, and daytime of EEG assessment were partialled out. FDR: False Discovery Rate according to Benjamini and Hochberg; BF_{10} Bayes factor showing the likelihood ratio between the alternate and null hypothesis (1/3 beta prior width).

* $p < .05$ (two-tailed nominal significance)

** FDR $< .05$ (p-value corrected for all tested associations using FDR method)

Table S5 Spearman correlations between NEO personality facets (T-Scores) and EEG-vigilance variables

$N = 468$	Mean vigilance				Stability score				Slope index			
	r_S	p	FDR	BF_{10}	r_S	p	FDR	BF_{10}	r_S	p	FDR	BF_{10}
Neuroticism												
N1 Anxiety	-.049	.290	.512	0.19	-.029	.533	.768	0.13	-.020	.667	.854	0.12
N2 Angry Hostility	-.054	.243	.455	0.21	-.028	.544	.768	0.13	.004	.928	.971	0.11
N3 Depression	-.007	.885	.949	0.11	.007	.877	.949	0.11	.022	.638	.844	0.12
N4 Self-Consciousness	-.068	.141	.327	0.32	-.021	.648	.845	0.12	.008	.868	.949	0.11
N5 Impulsiveness	-.150	.001*	.050**	19.88	-.076	.101	.276	0.41	-.087	.059	.203	0.63
N6 Vulnerability	.017	.707	.871	0.12	.009	.850	.949	0.11	.063	.172	.353	0.27
Extraversion												
E1 Warmth	-.116	.012*	.088	2.41	-.097	.036*	.165	0.95	-.119	.010*	.088	2.90
E2 Gregariousness	-.019	.677	.854	0.12	.007	.874	.949	0.11	-.005	.917	.971	0.11
E3 Assertiveness	-.081	.079	.237	0.50	-.083	.073	.229	0.53	-.109	.018*	.103	1.73
E4 Activity	-.102	.028*	.138	1.19	-.079	.090	.260	0.45	-.114	.014*	.090	2.14
E5 Excitement-Seeking	-.024	.607	.828	0.12	-.065	.158	.340	0.29	-.037	.430	.675	0.15
E6 Positive Emotions	-.083	.074	.229	0.53	-.065	.159	.340	0.29	-.116	.012*	.088	2.43
Openness												
O1 Fantasy	-.084	.071	.229	0.54	-.077	.095	.268	0.43	-.094	.041*	.168	0.85
O2 Aesthetics	-.092	.046*	.173	0.77	-.097	.037*	.165	0.93	-.137	.003*	.050**	8.60
O3 Feelings	-.108	.019*	.103	1.61	-.093	.044*	.172	0.80	-.137	.003*	.050**	8.27
O4 Actions	-.034	.457	.697	0.14	-.075	.107	.284	0.39	-.109	.019*	.103	1.68
O5 Ideas	-.068	.142	.327	0.31	-.095	.040*	.168	0.87	-.128	.006*	.073	4.76
O6 Values	.039	.406	.664	0.15	.014	.759	.923	0.11	-.002	.971	.980	0.11
Agreeableness												
A1 Trust	-.069	.133	.327	0.33	-.029	.536	.768	0.13	-.069	.138	.327	0.32
A2 Straightforwardness	-.002	.957	.979	0.11	-.013	.783	.931	0.11	.009	.841	.949	0.11
A3 Altruism	-.001	.980	.980	0.11	-.037	.428	.675	0.15	-.055	.239	.455	0.21
A4 Compliance	.050	.285	.512	0.19	.040	.387	.656	0.16	.029	.526	.768	0.13
A5 Modesty	.054	.240	.455	0.21	.088	.057	.203	0.65	.072	.118	.302	0.36
A6 Tender-Mindedness	-.146	.002*	.050**	15.16	-.119	.010*	.088	2.90	-.145	.002*	.050**	14.40
Conscientiousness												
C1 Competence	-.047	.312	.540	0.18	-.027	.566	.783	0.13	-.066	.157	.340	0.29
C2 Order	.012	.801	.936	0.11	.022	.635	.844	0.12	-.013	.787	.931	0.11
C3 Dutifulness	-.036	.435	.675	0.15	-.011	.819	.945	0.11	-.039	.403	.664	0.15
C4 Achievement Striving	-.115	.013*	.088	2.33	-.115	.013*	.088	2.32	-.135	.003*	.050**	7.65
C5 Self-Discipline	-.019	.684	.854	0.12	.003	.943	.975	0.11	-.028	.546	.768	0.13
C6 Deliberation	.064	.165	.345	0.28	.061	.188	.376	0.25	.049	.289	.512	0.19

FDR: False Discovery Rate according to Benjamini and Hochberg; BF_{10}: Bayes factor showing the likelihood ratio between the alternate and null hypothesis (1/3 beta prior width)

* $p < .05$ (two-tailed nominal significance)
** FDR $< .05$ (p-value corrected for all tested associations using FDR method)

Table S6 Partial Spearman correlations between NEO personality facets (T-Scores) and EEG-vigilance variables

$N = 468$	Mean vigilance				Stability score				Slope index			
	r_S	p	FDR	BF_{10}	r_S	p	FDR	BF_{10}	r_S	p	FDR	BF_{10}
Neuroticism												
N1 Anxiety	-.046	.326	.652	0.17	-.025	.596	.840	0.12	-.017	.716	.923	0.12
N2 Angry Hostility	-.055	.238	.531	0.22	-.028	.554	.830	0.13	.000	.994	.994	0.11
N3 Depression	-.011	.820	.951	0.11	.004	.937	.984	0.11	.015	.743	.941	0.11
N4 Self-Consciousness	-.072	.123	.429	0.35	-.026	.582	.840	0.13	.002	.962	.984	0.11
N5 Impulsiveness	-.146	.002*	.126	15.46	-.068	.143	.429	0.31	-.079	.089	.427	0.46
N6 Vulnerability	.018	.693	.917	0.12	.007	.874	.959	0.11	.060	.194	.527	0.25
Extraversion												
E1 Warmth	-.083	.075	.419	0.53	-.057	.219	.527	0.23	-.075	.108	.427	0.39
E2 Gregariousness	.010	.835	.951	0.11	.041	.381	.715	0.16	.032	.488	.813	0.14
E3 Assertiveness	-.070	.131	.429	0.34	-.068	.141	.429	0.32	-.095	.041*	.311	0.86
E4 Activity	-.085	.066	.398	0.58	-.057	.222	.527	0.23	-.087	.061	.390	0.62
E5 Excitement-Seeking	-.007	.877	.959	0.11	-.048	.302	.632	0.18	-.012	.798	.951	0.11
E6 Positive Emotions	-.058	.216	.527	0.23	-.034	.469	.797	0.14	-.074	.109	.427	0.39
Openness												
O1 Fantasy	-.070	.129	.429	0.34	-.063	.178	.515	0.27	-.076	.101	.427	0.41
O2 Aesthetics	-.076	.101	.427	0.41	-.079	.091	.427	0.45	-.118	.011*	.149	2.78
O3 Feelings	-.081	.080	.423	0.50	-.062	.183	.515	0.26	-.101	.030*	.242	1.14
O4 Actions	-.017	.718	.923	0.12	-.056	.231	.531	0.22	-.090	.053	.368	0.69
O5 Ideas	-.072	.119	.429	0.36	-.102	.027*	.242	1.23	-.135	.004*	.126	7.27
O6 Values	.030	.524	.827	0.13	.002	.967	.984	0.11	-.011	.818	.951	0.11
Agreeableness												
A1 Trust	-.058	.214	.527	0.23	-.015	.754	.943	0.11	-.053	.255	.547	0.21
A2 Straightforwardness	-.008	.865	.959	0.11	-.021	.659	.896	0.12	.002	.973	.984	0.11
A3 Altruism	.010	.823	.951	0.11	-.026	.572	.840	0.13	-.037	.430	.764	0.15
A4 Compliance	.041	.382	.715	0.16	.028	.551	.830	0.13	.011	.809	.951	0.11
A5 Modesty	.038	.419	.764	0.15	.070	.134	.429	0.33	.054	.242	.531	0.21
A6 Tender-Mindedness	-.128	.006*	.126	4.93	-.101	.029*	.242	1.14	-.124	.007*	.133	3.84
Conscientiousness												
C1 Competence	-.046	.326	.652	0.17	-.023	.623	.862	0.12	-.058	.214	.527	0.23
C2 Order	-.007	.884	.959	0.11	.002	.967	.984	0.11	-.028	.551	.830	0.13
C3 Dutifulness	-.030	.518	.827	0.13	-.002	.968	.984	0.11	-.020	.667	.896	0.12
C4 Achievement Striving	-.116	.012*	.149	2.51	-.115	.013*	.149	2.28	-.132	.005*	.126	5.98
C5 Self-Discipline	-.031	.506	.827	0.13	-.010	.834	.951	0.11	-.036	.433	.764	0.15
C6 Deliberation	.041	.381	.715	0.16	.034	.460	.796	0.14	.025	.598	.840	0.12

Effects of sex, age, and daytime of EEG-assessment were partialled out. FDR: False Discovery Rate according to Benjamini and Hochberg; BF_{10}: Bayes factor showing the likelihood ratio between the alternate and null hypothesis (1/3 beta prior width)

* $p < .05$ (two-tailed nominal significance)
